# Alterations in PTEN and *PIK3CA *in colorectal cancers in the EPIC Norfolk study: associations with clinicopathological and dietary factors

**DOI:** 10.1186/1471-2407-11-123

**Published:** 2011-04-07

**Authors:** Adam Naguib, James C Cooke, Lisa Happerfield, Lucy Kerr, Laura J Gay, Robert N Luben, Richard Y Ball, Panagiota N Mitrou, Alison McTaggart, Mark J Arends

**Affiliations:** 1 Medical Research Council Dunn Human Nutrition Unit, Wellcome Trust/MRC Building, Cambridge, CB2 0XY, UK; 2 Department of Pathology, University of Cambridge, Addenbrooke's Hospital, Cambridge CB2 0QQ, UK; 3 Medical Research Council Centre for Nutritional Epidemiology in Cancer Prevention and Survival, Department of Public Health and Primary Care, University of Cambridge, Cambridge CB1 8RN, UK; 4 Norfolk and Waveney Cellular Pathology Network, Norfolk and Norwich University Hospital NHS Foundation Trust, Colney Lane, Norwich, NR4 7UY, UK

## Abstract

**Background:**

The *PTEN *tumour suppressor gene and *PIK3CA *proto-oncogene encode proteins which contribute to regulation and propagation of signal transduction through the PI3K/AKT signalling pathway. This study investigates the prevalence of loss of PTEN expression and mutations in both *PTEN *and *PIK3CA *in colorectal cancers (CRC) and their associations with tumour clinicopathological features, lifestyle factors and dietary consumptions.

**Methods:**

186 adenocarcinomas and 16 adenomas from the EPIC Norfolk study were tested for *PTEN *and *PIK3CA *mutations by DNA sequencing and PTEN expression changes by immunohistochemistry. Dietary and lifestyle data were collected prospectively using seven day food diaries and lifestyle questionnaires.

**Results:**

Mutations in exons 7 and 8 of *PTEN *were observed in 2.2% of CRC and PTEN loss of expression was identified in 34.9% CRC. Negative PTEN expression was associated with lower blood low-density lipoprotein concentrations (p = 0.05). *PIK3CA *mutations were observed in 7% of cancers and were more frequent in CRCs in females (p = 0.04). Analysis of dietary intakes demonstrated no link between PTEN expression status and any specific dietary factor. PTEN expression negative, proximal CRC were of more advanced Dukes' stage (p = 0.02) and poor differentiation (p < 0.01). Testing of the prevalence of *PIK3CA *mutations and loss of PTEN expression demonstrated that these two events were independent (p = 0.55).

**Conclusion:**

These data demonstrated the frequent occurrence (34.9%) of PTEN loss of expression in colorectal cancers, for which gene mutations do not appear to be the main cause. Furthermore, dietary factors are not associated with loss of PTEN expression. PTEN expression negative CRC were not homogenous, as proximal cancers were associated with a more advanced Dukes' stage and poor differentiation, whereas distal cancers were associated with earlier Dukes' stage.

## Background

The PI3K/AKT signalling pathway affects many cellular processes including cell proliferation, apoptosis and invasion [1]. Signal transduction through this pathway is mediated through conversion of phosphatidylinositol bisphosphate (PIP_2_) to phosphatidylinositol triphosphate (PIP_3_) by phosphatidylinositol 3 kinases (PI3K) following their activation, and this reaction is antagonised by phosphatase and tensin homolog, deleted on chromosome ten (PTEN) activity. Of the genes which encode the enzymatic subunit of PI3K heterodimers, the *PIK3CA *gene, encoding the p110∝ protein, has been found to be most frequently, in not exclusively, mutationally activated in some human cancers [1,2]. In colorectal cancer (CRC), *PIK3CA *activating mutations have been described at frequencies of 10-20% [3-6], with two distinct regions, the helical and kinase domains, harbouring up to 80% of mutations [7].

The prevalence of *PTEN *mutations in CRC has been reported to vary between 1% and 29% [8-13]. This variability in observed *PTEN *mutation frequencies relates to tumour genomic instability, with *PTEN *mutations having been described in 14-30% of CRC with microsatellite instability (MSI-H) [9,14,15], but at very low frequencies (<5%) in unselected CRC [13]. Exons 7 and 8 of *PTEN *have been described to acquire more mutations than other regions of the gene in CRC, with insertions and deletions of adenine bases in poly-A tracts present in these exons being the predominant genetic change, consistent with frequent changes in repetitive sequences in MSI-H CRC [15]. Loss of PTEN expression has been reported at higher frequencies than mutation [12,15] with approximately 20-40% of CRC exhibiting loss of PTEN expression [16,17].

Incidence rates of colorectal cancer can vary up to 25-fold between countries [18] and it has been postulated that approximately 80% of observed national differences in incidence between can be attributed to dietary factors [19]. Although analysis of dietary components has been performed in relation to general colorectal cancer incidence, their exact relation to specific tumour suppressor gene losses and signalling pathway alterations remains to be fully investigated. To date, analysis of dietary factors in relation to PI3K/AKT pathway component changes in CRC has not been undertaken and little data exists describing the type of CRC in which *PIK3CA *oncogenic activations and PTEN inactivation occurs.

The present study aimed to investigate the relationship between *PTEN *and *PIK3CA *mutations and loss of PTEN expression in 186 colorectal adenocarcinomas from the EPIC Norfolk cohort and clinicopathological features, lifestyle traits and dietary factors, as well as analysing PTEN expression negative CRC stratified by stage and tumour location.

## Methods

### Study population, microsatellite instability analysis, dietary and lifestyle assessment

Exact descriptions of the study population, case ascertainment, methodology pertaining to dietary and lifestyle data acquisition and microsatellite instability status assessment have been described in detail elsewhere [20].

### Tissue processing and DNA extraction

Formalin fixed, paraffin embedded human tissue samples, biopsied from the caecum, proximal colon, distal colon and rectum, were processed for DNA extraction. Ten, 4 μm sections were cut from each block using a Microm HM 325 microtome (Thermo Scientific, Basingstoke, UK). A single section of tissue from each block was stained and used as a template for identification of adenocarcinoma, adenoma and normal tissue regions within each sample. To stain, sections underwent four, 15 minute washes: two in xylene followed by 2 in 100% ethanol. Four, 5 minute sequential washes in 95%, 80%, 70% and 40% ethanol, followed by rinsing in deionised water, completed the rehydration of the section. The sections were then immersed in Harris Haematoxylin Solution (Sigma-Aldrich, Gillingham, UK) for 30 seconds. Sections were then rinsed in tap water and stained with 1% Eosin Solution (Solmedia, Romford, UK) for 5 seconds. Following a final rinse with tap water to remove excess stain, cover slips were mounted using DePeX Mounting Medium Gurr (BDH Laboratory Supplies, Lutterworth, UK). Template slides were analysed by a consultant gastrointestinal histopathologist (MJA) using a light microscope and tissue regions corresponding to normal/tumour regions were identified and marked on the slide. Subsequently, the remaining 9 slides were dehydrated and cells from normal, adenoma or adenocarcinoma regions were carefully microdissected using a sterile scalpel. Tissue was collected starting 2 mm away from the normal/tumour tissue boundary, as indicated on the template slide, in order to minimize collection of non-tumour tissue in adenoma or adenocarcinoma samples.

### DNA extraction

Isolated tissue was digested in 240 μl of Buffer PKD with 10 μl of Proteinase K (both obtained from RNEasy kits, QIAGEN, Valencia, USA). Samples were agitated at 150 rpm at 55°C for 4-6 days with the level of tissue digestion checked after 3 days: those which still had visible amounts of tissue had a further 10 μl of Proteinase K added for the remainder of the incubation. Samples were then incubated at 80°C for 15 minutes in order to partially reverse formaldehyde modification of the nucleic acids and to denature any residual protein. These samples were then used directly, without further purification, for PCR amplification.

### Mutation detection

*PTEN *exons 7 and 8 were amplified using previously described primers [10]. PCR products were generated using 5 ng-2 μg of template DNA. KOD Hot Start DNA Polymerase kits (Novagen, Madison, USA) were used to make the following reaction mixture: 2.5 μl ×10 PCR Buffer for KOD Hot Start DNA Polymerase, 1 μl primers, forward and reverse (10 μM each), 1 μl MgSO_4 _(25 mM), 2 μl dNTPs (2 mM each), 0.25 μl KOD DNA Polymerase and made up to a total reaction volume of 25 μl with nuclease free water (Promega, Madison, USA). The reactions involved a denaturation step at 94°C for 5 minutes followed by 45 cycles of 94°C for 15 seconds, then 30 seconds at annealing temperatures of either 56°C or 54°C for *PTEN *exons 7 or 8 respectively, followed by extension at 72°C for 30 seconds. Lastly, a final extension step of 72°C for 5 minutes was performed. *PIK3CA *exon 8 was amplified using primers 8F (5'-CAT AAA TTA GAT ATT TTT TAT GGC AGT CAA AC-3') and 8R (5'-GAG AAA GTA TCT ACC TAA ATC CAC AGA TTA TAA TTG-3'). *PIK3CA *exon 9 was amplified using primers 9F (5'-TTG CTT TTT CTG TAA ATC ATC TGT G-3') and 9R (5'-CTG CTT TAT TTA TTC CAA TAG GTA TG-3'). *PIK3CA *exon 20 was amplified using previously described primers [21]. The PCR was as described above for *PTEN *except that annealing temperatures of 55°C were used for amplification of all three *PIK3CA *exons. To detect successful amplifications, 5 μl of each reaction mixture was separated on a 1.5% agarose gel containing 1 μg/ml ethidium bromide, and visualised under UV light. The remaining PCR amplification product mixture (20 μl) following visualisation on agarose gels was purified using Multiscreen filter plates (Millipore, Billerica, USA) according to the manufacturer's instructions and subjected to direct sequencing by ABI3730xl Platform sequencer (Applied Biosystems, Warrington, UK). Forward and reverse strands were both sequenced. Every sample was PCR amplified and sequenced independently a minimum of twice on each strand.

### PTEN immunohistochemistry

PTEN protein immunohistochemistry (IHC) was performed using the monoclonal antibody 6H2.1 (Cascade Biosciences, Winchester, USA). Blocking, addition of secondary antibody and washing reactions were performed according to the manufacturer's instructions for the BondMax staining system with Bond Polymer Refine Detection Reagents (Leica Microsystems, Wetzlar, Germany). Antigen retrieval was performed for 30 minutes at 98°C in 0.01 mol/L sodium citrate buffer at pH 6.4. Primary antibody was applied for 30 minutes at a 1:100 dilution. Secondary antibody was anti-mouse IgG-HRP (8 μg/ml) and was applied for 15 minutes.

PTEN protein expression was classified as negative if over 50% of the tumour cells present demonstrated loss of expression. In order to assess PTEN expression status in cancer cells, the PTEN expression levels in normal mucosa on the same slide were used as a reference. Due to the difference in PTEN staining patterns between stromal fibroblasts, lymphoid cells and endothelial cells in the gut, these stromal cells were used only as an internal control to assess successful IHC staining, not as a reference for comparison with cancer cell PTEN expression levels. Instead, non-cancerous mucosa were used to assess loss of PTEN expression in neoplastic crypts.

### Statistical analysis

Analysis of lifestyle and dietary factors, clinicopathological cancer features and patient characteristics was performed using chi-squared (χ^2^) tests for categorical data and analysis of variance (ANOVA) tests for continuous numerical data. For these analyses, tumour samples were classified as PTEN expression positive or negative and *PIK3CA *wildtype or mutated (in exons 8, 9 or 20). For additional testing, PTEN negative adenocarcinomas were classified by both Dukes' stage (early: Dukes' stages A or B; advanced: Dukes' stages C or D) and independently by tumour location (proximal colonic up to the splenic flexure, or distal colonic/rectal) and tested for association with dietary, lifestyle and clinicopathological factors. Differentiation was determined for all cases by a consultant histopathologist (MJA) and classified as moderately/well differentiated or poorly differentiated and cancers were classified as demonstrating either microsatellite instability (MSI) or microsatellite stable (MSS) status. Lifestyle factors, including smoking status (current/former/never), physical activity (high/low), alcohol consumption (g/day, continuous), low-density lipoprotein (mmol/l, continuous) and high-density lipoprotein (mmol/l, continuous) blood concentrations, triglyceride blood concentrations (mmol/l, continuous) and plasma vitamin C concentrations (μmol/l, continuous) were also tested for association with loss of PTEN expression and *PIK3CA *mutations. Continuous dietary variables were tested for association with PTEN expression status including meat, fruit and vegetables, fat, vitamin, fibre and macronutrients, including calcium, in their relevant unit of consumption. Additionally, PTEN expression negative adenocarcinomas, categorised by location and Dukes' stage, were also tested for associations with dietary factors. A probability value of less than or equal to 0.05 was considered to be statistically significant. No adjustment was made for multiple testing. All statistical testing was undertaken using SPSS version 16.0 (SPSS Inc, Chicago, USA).

## Results

### *PTEN *and *PIK3CA *mutation frequencies and PTEN expression status in colorectal adenocarcinomas and adenomas

**Figure 1:**
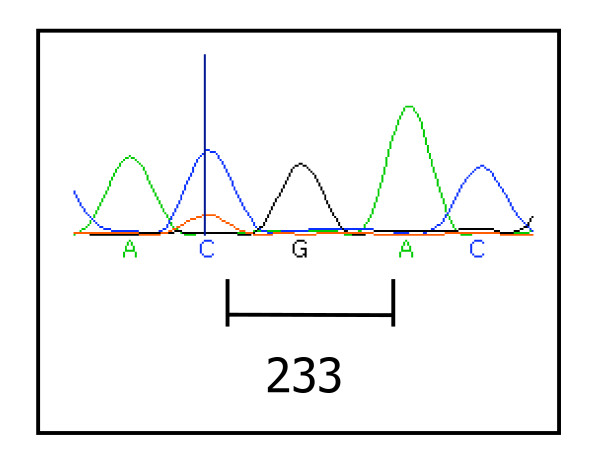
**Mutation observed in *PTEN *exon 7**. Codon 233 encoding arginine (CGA), is changed to a stop codon (TGA), resulting in truncation of the PTEN protein.

**Figure 2:**
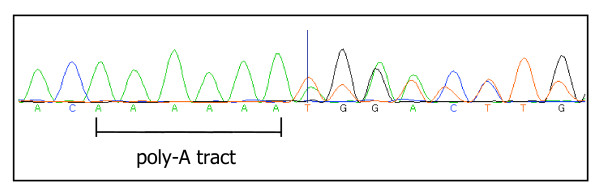
**Single base insertion in the poly-A tract of *PTEN *exon 8**. A frameshift mutation in the *PTEN *gene caused by the single insertion of an additional adenine base in the poly-A tract of exon 8.

The type and distribution of the mutations observed in *PTEN *and *PIK3CA *are described in Table 1. Of the 186 adenocarcinoma samples analysed, 4 (2.2%) harboured *PTEN *mutations in exons 7 or 8. One sample had mutations in both exons 7 and 8. Three samples showed mutations in exon 8 only. The mutation in exon 7 was a C to T transition which resulted in codon 233 of the protein, arginine (CGA), being converted to a stop codon (TGA) (Figure 1). This same cancer had a mutation in exon 8: a C to T transition changing codon 339 (CCA) phenylalanine to serine (TCA). The remaining three cancers harboured changes in *PTEN *exon 8, all of which involved changes in the poly-A tract (codons 321-323). These were either a single base deletion or a single base insertion (Figure 2), shortening the tract from 6 to 5, or lengthening the tract from 6 to 7 adenine bases respectively, resulting in a coding frameshift. The final mutation observed in *PTEN *exon 8 was a 23 base deletion covering the poly-A tract. This deletion was from the first base of codon 316 to the second base of codon 323 inclusive, resulting in a coding frameshift. None of the 16 adenomas analysed contained mutations in *PTEN *exons 7 or 8.

**Table 1:** The type and distribution of mutations in *PTEN *exons 7 and 8 and *PIK3CA *exons 8, 9 and 20 in 186 adenocarcinoma and 16 adenoma samples available from EPIC Norfolk.

	*PTEN *mutations in exons 7 and 8		
	Mutations in adenocarcinomas	Mutations in adenomas	Total
Codon 233: wildtype CGA*			
TGA (Arg to Stop)	1	0	1

Codon 339: wildtype CCA*			
TCA (Phe to Ser)	1	0	1

**Poly-A tract (codons 321-323)**			
1 bp insertion	1	0	1

**Poly-A tract (codons 321-323)**			
1 bp deletion	1	0	1

**Codon 316 (1**^**st **^**base) to 323 (2**^**nd **^**base)**			
23 bp deletion	1	0	1

**Total**	5	0	**5**

	***PIK3CA *mutations in exons 8, 9 and 20**		
	Mutations in adenocarcinomas	Mutations in adenomas	Total

**Codon 542: wildtype GAA**			
GTA (Glu to Val)	1	0	1
AAA (Glu to Lys)	2	0	2

**Codon 545: wildtype GAG**			
AAG (Glu to Lys)	2	1	3

**Codon 546: wildtype CAG**			
AAG (Gln to Lys)	2	0	2
CGG (Gln to Arg)	1	0	1

**Codon 1046: wildtype CAT**			
CGT (His to Arg)	5	0	5

**Total**	13	1	**14**

*These two mutations were observed in the same adenocarcinoma.

Analysis of *PIK3CA *exons 8, 9 and 20 demonstrated that 13 adenocarcinomas (7.0%) harboured mutations (Table 1). None of the identified mutations were in *PIK3CA *exon 8. Eight cancer samples possessed mutations in exon 9 and 5 cancers showed mutations in exon 20. In addition to the five non-synonymous base changes observed in exon 20, four synonymous SNPs were also detected in this exon. All four were the same, previously reported, SNP (rs17849079) that changes codon 1025 to ACT instead of ACC: both codons encode threonine. Of the 16 adenoma samples tested for mutation, one (6.3%) harboured a mutation in *PIK3CA *in exon 9. This single *PIK3CA *mutated adenoma presented in an individual as an isolated neoplasm, without concurrent adenocarcinoma.

**Figure 3:**
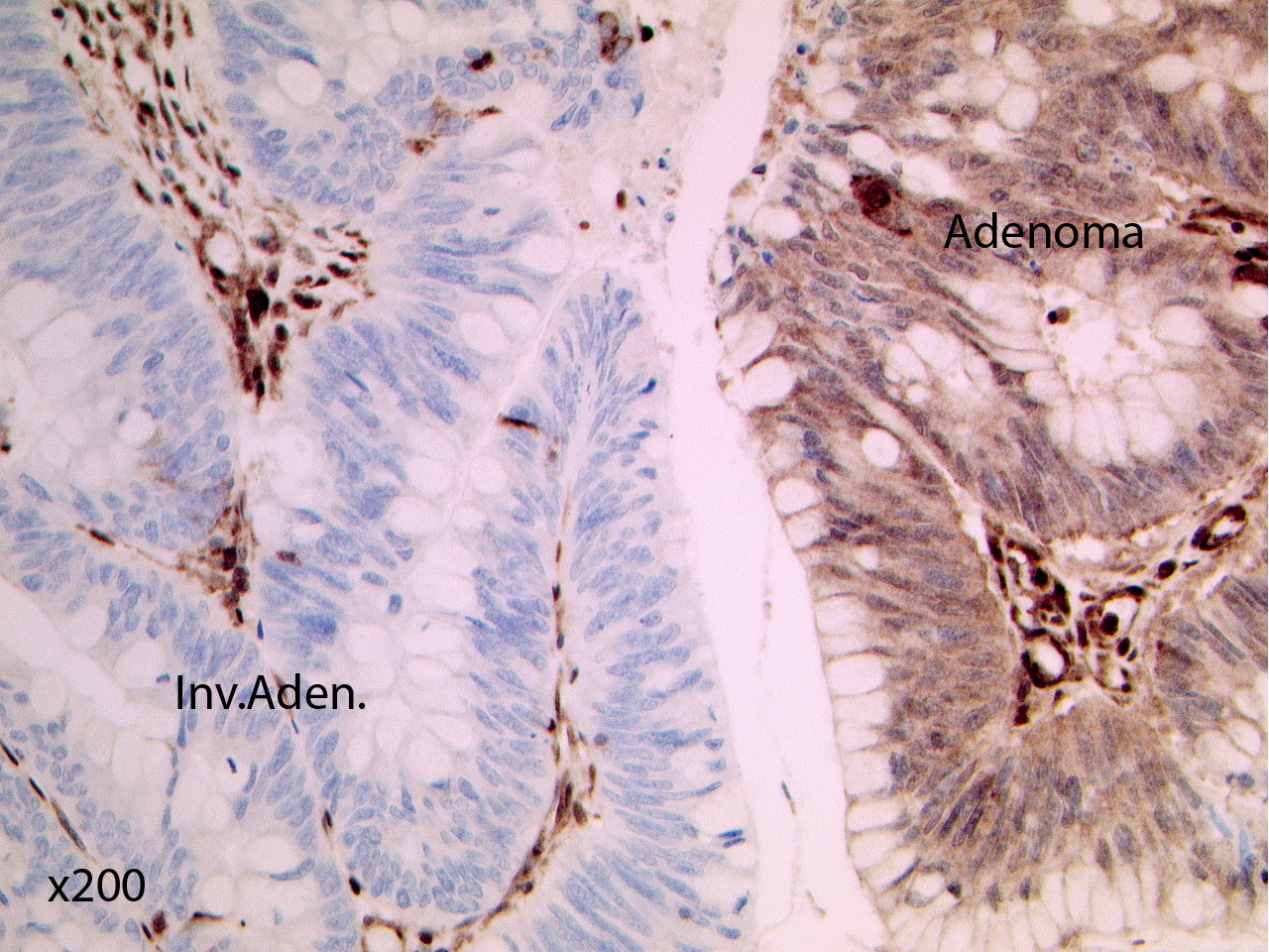
**PTEN immunohistochemistry of an invasive adenocarcinoma [Inv.Aden.] and adjacent adenoma [Adenoma] which demonstrates altered PTEN expression**. Both adenoma and adenocarcinoma are surrounded by lymphoid and stromal cells which are expressing PTEN. The normal stromal cells exhibit strong nuclear and cytoplasmic staining. The adenoma cells appear to have reduced nuclear but retained cytoplasmic staining, whereas the invasive cancer cells have lost both nuclear and cytoplasmic PTEN expression (×200).

**Figure 4:**
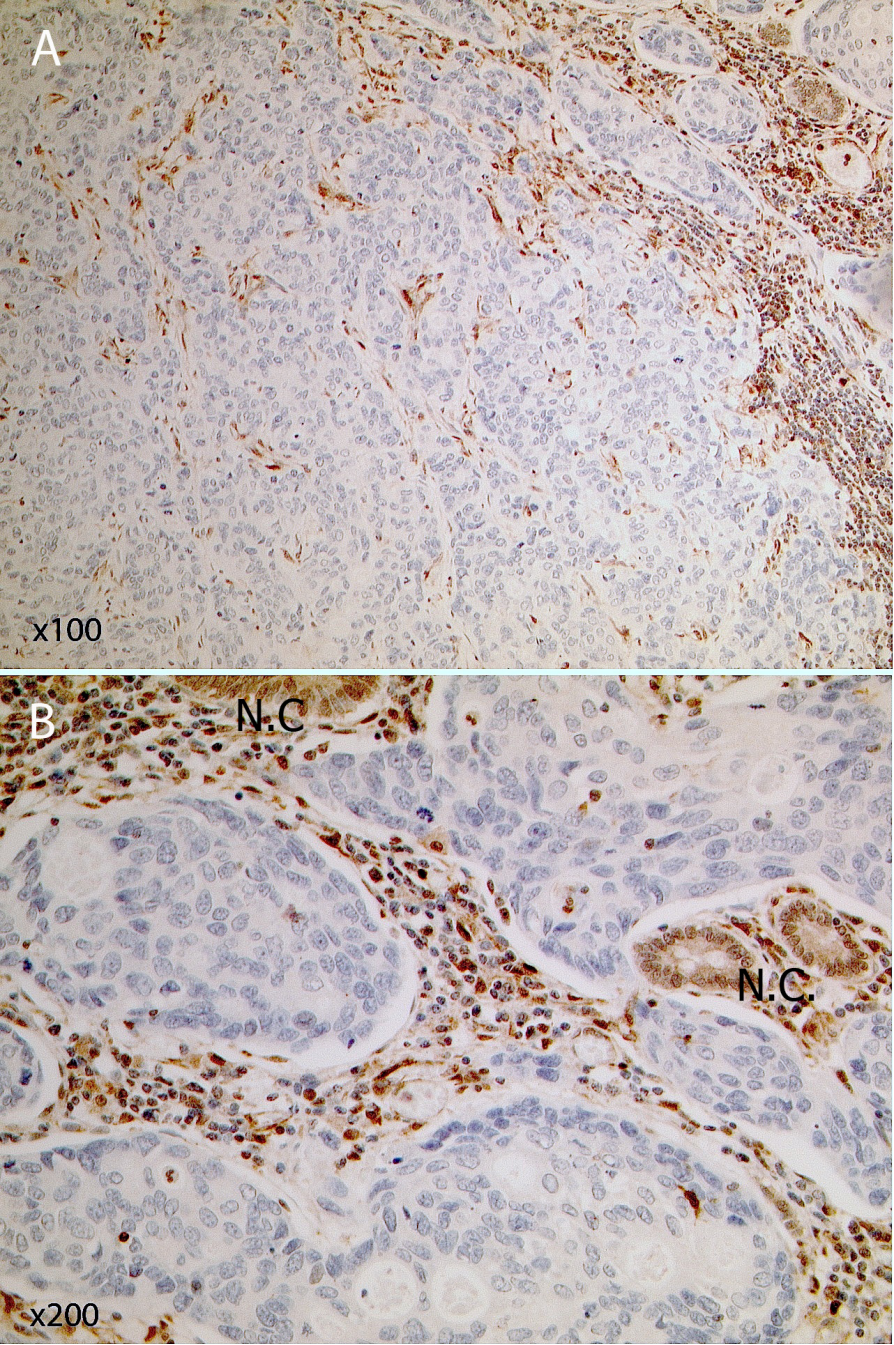
**Immunohistochemical analysis showing loss of PTEN expression in a colorectal adenocarcinoma**. A: Colorectal adenocarcinoma showing loss of PTEN expression by immunohistochemistry (×100). The cancer cells show lack of expression, whereas stromal lymphoid and fibroblastic cells demonstrate high levels of PTEN expression in the nucleus with reduced but evident cytoplasmic staining. B: A different region of the same adenocarcinoma magnified (×200) to reveal some foci of entrapped non-cancerous epithelial cells [N.C.] showing PTEN expression in both nuclear and cytoplasmic compartments with weaker expression in their nuclei comparative to the nuclei of lymphoid and stromal cells.

PTEN expression data were obtained for 172 adenocarcinomas. No data were obtained for 14 samples due to lack of tissue, poor sample fixation or non-interpretable immunohistochemical staining due to technical problems. Of these 172 samples, 60 (34.9%) demonstrated loss of PTEN expression (Figures 3 and 4). All four adenocarcinomas with *PTEN *mutations demonstrated loss of PTEN expression. Expression data were available for 14 adenoma samples, 2 (14.3%) of which demonstrated loss of PTEN expression. One of the adenomas demonstrating negative PTEN expression presented in isolation in the absence of an adenocarcinoma, whereas the second adenoma presented with a coincident adenocarcinoma. However, negative PTEN expression was only identified in the adenoma, not in the separate cancer obtained from the same individual.

### Lifestyle and clinicopathological factors in relation to PTEN expression and *PIK3CA *mutation

Analysis of the distribution of cancers with loss of PTEN expression and *PIK3CA *mutations according to clinicopathological and lifestyle variables is presented in Table 2. Stratification of clinicopathological and lifestyle variables by *PTEN *mutation status in isolation was not performed due to the small number of samples demonstrating a mutated *PTEN *genotype in exons 7 and 8. Individuals with cancers lacking PTEN expression exhibited significantly lower mean blood LDL concentrations than those with cancers demonstrating normal PTEN expression (p = 0.05): 3.90 mmol/l *versus *4.28 mmol/l. Testing of *PIK3CA *mutation status according to lifestyle and clinicopathological factors demonstrated an increased prevalence of *PIK3CA *mutations in females with CRC (p = 0.04).

**Table 2:** Clinicopathological and lifestyle characteristics of colorectal cancer cases by stratified PTEN expression and *PIK3CA *mutation status.

	PTEN expression status			*PIK3CA *mutation status		
Characteristic	Positive	Negative	***P***^**‡**^	Wildtype	Mutant	***P***^**‡**^
	***n *= 112**^**†**^	***n *= 60**^**†**^		***n *= 172**^**†**^	***n *= 13**^**†**^	
**Sex**						
Male	48.2 (54)	46.7 (28)		52.0 (90)	23.1 (3)	
Female	51.8 (58)	53.3 (32)	0.85	48.0 (83)	76.9 (10)	**0.04**
**Age at diagnosis **(years)	71.0 (7.5)	70.0 (8.0)	0.43*	70.3 (7.7)	73.3 (5.6)	0.17*
**Tumour location**						
Proximal colonic	34.0 (35)	42.9 (24)		34.4 (55)	53.8 (7)	*(FET)*
Distal colonic/Rectal	66.0 (68)	57.1 (32)	0.27	65.6 (105)	46.2 (6)	0.23
**Differentiation**						
Well/Moderate	86.9 (86)	87.0 (47)		84.9 (129)	92.3 (12)	*(FET)*
Poor	13.1 (15)	13.0 (7.0)	0.98	15.1 (23)	7.7 (1)	0.69
**Dukes' Stage**						
A/B	53.6 (52)	55.8 (29)		54.4 (80)	61.5 (8)	
C/D	46.4 (45)	44.2 (23)	0.80	45.6 (67)	38.5 (5)	0.62
**MSI status**						
MSS	83.0 (88)	83.3 (45)		84.6 (126)	63.6 (7)	*(FET)*
MSI	17.0 (18)	16.7 (9)	0.96	15.4 (23)	36.4 (4)	0.09
**BMI **(kg/m^2^)	27.3 (4.1)	26.6 (4.4)	0.36*	27.3 (4.3)	25.7 (4.4)	0.20*
**Alcohol consumption**	9.7 (16)	6.6 (9.7)	0.17*	9.5 (15.0)	5.4 (10)	0.34*
**Smoking status**						
Current	12.0 (13)	8.3 (5)		11.2 (19)	0.0 (0)	
Former	46.3 (50)	45.0 (27)		45.6 (77)	58.3 (7)	
Never	41.7 (45)	46.7 (28)	0.69	43.2 (73)	41.7 (5)	0.42
**Physical activity**						
Low	64.3 (72)	70.0 (42)		65.7 (113)	84.6 (11)	*(FET)*
High	35.7 (40)	30.0 (18)	0.45	34.3 (59)	15.4 (2)	0.23
**HRT status**						
Current	10.3 (6)	6.5 (2)		9.8 (8)	0.0 (0)	
Former	17.2 (10)	16.1 (5)		17.1 (14)	20.0 (2)	
Never	72.4 (42)	77.4 (24)	0.81	73.2 (60)	80.0 (8)	0.58
**LDL cholesterol **(mmol/l)	4.28 (1.2)	3.90 (1.1)	0.05*	4.12 (1.1)	4.30 (1.0)	0.61*
**HDL cholesterol **(mmol/l)	1.31 (0.4)	1.30 (0.4)	0.96*	1.30 (0.4)	1.29 (0.4)	0.93*
**Triglyceride **(mmol/l)	1.99 (0.8)	2.25 (1.6)	0.17*	2.09 (1.1)	2.11 (1.5)	0.95*
**Plasma vitamin C **(μmol/l)	50.3 (18)	53.3 (33)	0.47*	51.2 (24)	54.1 (19)	0.71*

^‡^P values determined by χ2 test or Fisher's exact test when one or more expected values were less than 5 (denoted by FET). *: ANOVA tests used to calculate p-values. Results presented as % (n) or mean (±SD). HRT: hormone replacement therapy. MSI: Microsatellite instability. MSS: Microsatellite stable. ^† ^Not all individuals had data for each variable, in these cases these individuals were omitted from the test. PTEN expression data were available for 172 cases. For HRT testing only females were analysed.

### Dietary factors and PTEN expression in CRC

None of the dietary variables tested displayed a statistically significant association with cancers positively or negatively expressing PTEN (Table 3). Testing of *PIK3CA *mutation status according to dietary consumptions was not performed due to the calculated low power of such testing as <10% of cases demonstrated mutations in the three *PIK3CA *exons analysed.

**Table 3:** Dietary intakes stratified by PTEN expression.

	PTEN expression status		
Dietary factor	Positive *n *= 112	Negative *n *= 60	***P***^***‡***^
**Meat**			
Red Meat (g/d)	36 (27.1)	39 (30.3)	0.56
Processed Meat (g/d)	26 (19.2)	24 (19.5)	0.60
Red + Processed Meat (g/d)	62 (37.3)	63 (34.1)	0.86
White Meat (g/d)	19 (18.7)	21 (18.2)	0.49
White Fish (g/d)	17 (19.5)	16 (14.0)	0.59
Fatty Fish (g/d)	12 (21.8)	10 (13.7)	0.38
**Fruit and vegetables**			
Fruit (g/d)	177 (144.1)	163 (125.0)	0.53
Vegetables (g/d)	135 (73.1)	140 (60.0)	0.63
**Fat**			
Total Fat (g/d)	70 (22.9)	73 (22.2)	0.31
PUFA (g/d)	13 (5.4)	14 (5.2)	0.50
MUFA (g/d)	24 (8.1)	26 (7.9)	0.12
SFA (g/d)	27 (10.3)	28 (9.8)	0.53
**Vitamins**			
B2[riboflavin] (mg/d)	2 (0.6)	2 (0.6)	0.91
B3[niacin] (mg/d)	18 (5.4)	18 (6.3)	0.56
B6[pyroxidine] (μg/d)	2 (0.6)	2 (0.6)	0.76
B9[folate] (μg/d)	257 (73.3)	260 (69.2)	0.79
B12 (μg/d)	6 (5.6)	5 (3.7)	0.29
C (mg/d)	85 (49.0)	84 (44.8)	0.85
D (μg/d)	3 (2.2)	3 (2.2)	0.99
**Fibre and Macronutrients**			
Total Energy (MJ/d)	8 (2.1)	8.0 (1.9)	0.38
Carbohydrate (g/d)	230 (65.4)	245 (67.9)	0.17
Protein (g/d)	70 (15.1)	70 (14.3)	0.97
NSP (g/d)	14 (5.7)	15 (5.3)	0.44
Calcium (mg/d)	773 (239.2)	795 (220.1)	0.55

^‡^P values determined by ANOVA. Results presented as mean (±SD). PUFA: polyunsaturated fatty acid. MUFA: monounsaturated fatty acid. SFA: saturated fatty acid. NSP: non-starch polysaccharide. PTEN expression data were available for 172 cases.

### PTEN expression negative cancers stratified by Dukes' stage and tumour location

In order to assess the significance of Dukes' stage and colorectal location relative to PTEN expression, cancers lacking PTEN expression were stratified by these characteristics (Table 4). Cancers demonstrating loss of PTEN expression and of a less advanced Dukes' stage (A/B) were more frequently located in the distal colon or rectum; whereas cancers demonstrating loss of PTEN expression and advanced Dukes' stage (C/D) were more frequently located in the proximal colon (p = 0.02). Additionally, cancers of all stages located in the proximal colon lacking PTEN expression were more frequently poorly differentiated than those that were in the distal colon or rectum (p < 0.01).

**Table 4:** Clinicopathological and lifestyle characteristics of PTEN expression negative colorectal cancers stratified by Dukes' stage and colorectal location.

	PTEN expression negative colorectal adenocarcinomas					
Characteristic	Dukes' stage A/B ***n *= 29**^**†**^	Dukes' stage C/D ***n *= 23**^**†**^	***P***^***‡***^	Proximal colonic ***n *= 24**^**†**^	Distal colonic/Rectal ***n *= 32**^**†**^	***P***^***‡***^
**Sex**						
Male	58.6 (17)	39.1 (9)		41.7 (10)	46.9 (15)	
Female	41.4 (12)	60.9 (14)	0.16	58.3 (14)	53.1 (17)	0.70
**Age at diagnosis **(years)	70.3 (8.14)	69.0 (8.67)	0.56	71.0 (7.31)	69.1 (8.77)	0.41
**Tumour location**						
Proximal colonic	37.5 (9)	72.0 (18)		^a^	^a^	
Distal colonic/Rectal	62.5 (15)	28.0 (7)	**0.02**	^a^	^a^	^a^
**Differentiation**						
Well/Moderate	92.6 (25)	78.3 (18)	*(FET)*	69.6 (16)	100.0 (28)	*(FET)*
Poor	7.4 (2)	21.7 (5)	0.23	30.4 (7)	0.0 (0)	**<0.01**
**Dukes' Stage**						
A/B	^a^	^a^		37.5 (9)	72.0 (18)	
C/D	^a^	^a^	^a^	62.5 (15)	28.0 (7)	**0.02**
**MSI status**						
MSS	81.5 (22)	84.2 (16)	*(FET)*	72.7 (16)	89.7 (26)	*(FET)*
MSI	18.5 (5)	15.8 (3)	1.00	27.3 (6)	10.3 (3)	0.15
**BMI **(kg/m^2^)	26.2 (3.60)	26.4 (4.77)	0.85	26.5 (4.71)	26.4 (3.64)	0.93
**Alcohol consumption**	6.6 (11.1)	6.5 (7.8)	0.97	4.0 (5.7)	8.0 (10.8)	0.11
**Smoking status**						
Current	13.8 (4)	4.3 (1)		8.3 (2)	9.4 (3)	
Former	41.4 (12)	52.2 (12)		54.2 (13)	40.6 (13)	
Never	44.8 (13)	43.5 (10)	0.47	37.5 (9)	50.0 (16)	0.60
**Physical activity**						
Low	69.0 (20)	65.2 (15)		58.3 (14)	78.1 (25)	
High	31.0 (9)	34.8 (8)	0.78	41.7 (10)	21.9 (7)	0.11
**LDL cholesterol **(mmol/l)	3.70 (1.05)	3.94 (1.11)	0.45	3.82 (1.17)	3.97 (0.98)	0.62
**HDL cholesterol **(mmol/l)	1.29 (0.39)	1.33 (0.50)	0.76	1.30 (0.51)	1.34 (0.36)	0.69
**Triglyceride **(mmol/l)	1.90 (1.21)	2.75 (1.95)	*0.06*	2.48 (1.61)	1.97 (1.35)	0.21
**Plasma vitamin C **(μmol/l)	46.6 (18.9)	52.7 (23.1)	0.32	48.1 (19.5)	56.6 (40.1)	0.37

^‡^P values determined by χ2 test or Fisher's exact test when one or more expected values were less than 5 (denoted by FET). *: ANOVA tests used to calculate p-values. Results presented as % (n) or mean (±SD). HRT: hormone replacement therapy. MSI: Microsatellite instability. MSS: Microsatellite stable. ^† ^Not all individuals had data for each variable, in these cases these individuals were omitted from the test. HRT status was not analysed due to the low numbers of cases available for testing. ^a ^Testing of Dukes' stage in relation to cancer location in the bowel is presented once per test set.

All dietary factors were tested for association with PTEN expression negative cancers stratified by Dukes' stage and colorectal location. Advanced stage (Dukes' C and D) cancers demonstrating loss of PTEN expression were associated with a higher consumption of non-starch polysaccharides than those PTEN expression negative cancers which were of a less advanced stage (Dukes' A and B) (p = 0.01). No other dietary variables tested displayed a statistically significant association with either early or advanced Dukes' stage, PTEN expression negative cancers or proximal colonic or distal colonic/rectally located PTEN negative cancers.

### Mutations in *PTEN *and *PIK3CA *and loss of PTEN expression in relation to *BRAF *and *K-RAS *mutations

In a previous analysis performed in our laboratory, the 186 colorectal adenocarcinomas and 16 adenomas described in this report were tested for the presence of *BRAF *and *K-RAS *mutations [20]. In order to assess any relationship between oncogenic activation or loss of tumour suppressor function in the PI3K/AKT and MAPK/ERK signalling pathways, mutations in *PIK3CA*, *PTEN*, *BRAF *and *K-RAS*, as well as loss of PTEN expression, were tested in relation to each other (Table 5). Our previous analyses demonstrated that within the 186 adenocarcinomas tested, *BRAF *and *K-RAS *mutations occurred together less frequently than expected by chance (χ^2 ^test, p = 0.009). Further analysis demonstrated that *PTEN *exon 7 and 8 and *PIK3CA *exon 8, 9 and 20 mutations were not related in either a mutually exclusive or a co-occurring manner (Fisher's exact test, p = 0.25). *PIK3CA *mutations and PTEN loss of expression were also not related (Fisher's exact test, p = 0.55). Further tests showed that *PTEN *mutations and *BRAF *mutations were associated (Fisher's exact test, p < 0.001) and both were associated with MSI (*BRAF*: p < 0.001, *PTEN*: Fisher's exact test p = 0.01). Of the four adenocarcinomas which exhibited mutations in *PTEN *exons 7 and 8, all also harboured *BRAF *mutations. Analysis of PTEN loss of expression and *BRAF *mutations did not demonstrate co-occurrence (χ^2 ^test, p = 0.80). *K-RAS *mutations were not linked to either mutations of either *PIK3CA *or *PTEN *or loss of PTEN expression. Due to the low number of adenomas available for analysis, testing for the co-incidence of mutations in several genes was not performed these pre-cancerous lesions.

**Table 5:** The distribution of *PTEN*, *PIK3CA*, *BRAF *and *K-RAS *mutations and loss of PTEN expression in the 186 adenocarcinomas available from EPIC Norfolk.

	*BRAF *mutant	*BRAF *wildtype	
*K-RAS *mutant	3.4 (1)	25.5 (40)	
*K-RAS *wildtype	96.6 (28)	74.5 (117)	p = 0.009
	*BRAF *mutant	*BRAF *wildtype	
*PTEN *mutant	**13.8 (4)**	**0.0 (0)**	(FET)
*PTEN *wildtype	**86.2 (25)**	**100.0 (157)**	**p < 0.001**

	*BRAF *mutant	*BRAF *wildtype	
PTEN expression negative	37.0 (10)	34.5 (50)	
PTEN expression positive	63.0 (17)	65.5 (95)	p = 0.80

	*BRAF *mutant	*BRAF *wildtype	
*PIK3CA *mutant	13.8 (4)	5.7 (9)	(FET)
*PIK3CA *wildtype	86.2 (25)	94.3 (148)	p = 0.12

	*K-RAS *mutant	*K-RAS *wildtype	
*PIK3CA *mutant	7.3 (3)	6.9 (10)	(FET)
*PIK3CA *wildtype	92.7 (38)	93.1 (135)	p = 1.00

	*K-RAS *mutant	*K-RAS *wildtype	
*PTEN *mutant	0.0 (0)	2.8 (4)	(FET)
*PTEN *wildtype	100.0 (41)	97.2 (141)	p = 0.58

	*K-RAS *mutant	*K-RAS *wildtype	
PTEN expression negative	41.7 (15)	33.1 (45)	
PTEN expression positive	58.3 (21)	66.9 (91)	p = 0.34

	*PIK3CA *mutant	*PIK3CA *wildtype	
PTEN expression negative	23.1 (3)	35.8 (57)	(FET)
PTEN expression positive	76.9 (10)	64.2 (102)	p = 0.55

	*PIK3CA *mutant	*PIK3CA *wildtype	
*PTEN *mutant	7.7 (1)	1.7 (3)	(FET)
*PTEN *wildtype	92.3 (12)	98.3 (170)	p = 0.25

P values determined by χ2 test or Fisher's exact test when one or more expected values were less than 5 (denoted by FET). Results presented as % (n). PTEN expression data were not available for 12/186 colorectal adenocarcinomas: these cases were omitted from testing.

## Discussion

The data presented confirm that loss of PTEN expression occurs in a significant proportion (35%) of colorectal cancers and may be due to mutation in only a minority of cases. Loss of PTEN expression has previously been reported at higher rates than mutation in colorectal cancer [12,15]. Loss of PTEN expression is associated with different clinicopathological features, including cancer stage and position in the bowel. However, loss of PTEN expression does not appear to be associated with any particular dietary factors. Mutations in *PIK3CA *were observed at a lower prevalence (7%) than loss of PTEN expression.

Our observation of approximately one third of cancers demonstrating loss of PTEN expression is in keeping with other reports which have identified 20-40% of CRC as PTEN expression negative [16,17], as is our observation of low *PTEN *mutation rates that have been reported in previous studies. One study of 72 unselected colorectal cancers analysing all 9 exons of *PTEN *identified a mutation frequency of 1.4% [13]. Two studies, both analysing 32 microsatellite unstable colorectal cancers, identified *PTEN *mutation frequencies of 18.8% [14] and 14.0% [15] in MSI+ CRC. As approximately 15% of all colorectal cancers display microsatellite instability [22], these data suggest that in an unselected sample set, if *PTEN *mutation is found predominantly or entirely in MSI+ colorectal cancers, approximately 2-3% of all colorectal cancer samples may be expected to exhibit *PTEN *mutations. Our data corroborate this estimation.

Of the adenocarcinomas tested in this report, 17.1% were classified as microsatellite unstable [20] and of these MSI+ cases, 4 (13.8%) harboured *PTEN *mutations, a figure consistent with previously published observations [14]. Statistical testing of the distribution of *PTEN *mutations and MSI demonstrated a positive association between these two features (FET, p = 0.01). Although testing of greater numbers of *PTEN *mutant samples is required to validate these observations, these data show that MSI and *PTEN *mutations, in exons 7 and 8 at least, occur together in the same colorectal cancers, along with *BRAF *mutations. This association is consistent with the type of *PTEN *mutations observed in our analyses. Three of the four observed mutations were repetitive sequence insertions or deletions involving the poly-A tracts in *PTEN*, consistent with mismatch repair deficiency, which is the cause of MSI in cancers. Loss of PTEN expression in general, unlike *PTEN *mutation, was not linked to MSI.

It has been reported that the majority of *PTEN *mutations in CRC may occur in exons 7 and 8 [15], which in our cohort harboured mutations in only 2.2% of CRC samples, in keeping with published studies [13]. Following this, we performed an immunohistochemical (IHC) analysis of PTEN protein expression as there are advantages of such an approach. First, IHC analysis of tissue samples circumvents the requirement for PCR based sequencing analyses of all 9 exons of *PTEN *or of the analysis of *PTEN *cDNA, which is identical to the non-translated transcript of the *PTEN *pseudogene except for 18 bp [23]. Second, expression analysis by IHC allows for all factors, including mutation, promoter methylation, miRNA alterations or *PTEN *gene copy number changes, that affect PTEN protein expression levels in colorectal tumours, to be assessed in one assay with direct comparison of normal, adenoma and adenocarcinoma cells. Third, this integrative assay identified a large enough group of CRCs with abnormal PTEN activity and presence to allow meaningful statistical testing of associations with dietary and lifestyle factors.

Loss of PTEN protein expression may be explained by several mechanisms in addition to mutation. CpG island methylator phenotype (CIMP), resulting in silencing of expression of affected genes occurs in 10-20% CRC [24]. Methylation at the *PTEN *locus (10q23) may be responsible for loss of expression observed in some of the CRC lacking *PTEN *mutations; an observation previously described in MSI CRC [11]. Additionally, chromosomal instability (CIN) leading to loss of heterozygosity (LOH) at 10q23 may also have contributed to the loss of PTEN expression observed, as LOH at 10q23 has been documented in 20-30% of colorectal cancers [12,15,25].

Analysis of clinicopathological cancer features and dietary and lifestyle factors relative to PTEN expression status demonstrated no associations with PTEN expression except in the case of blood low-density lipoprotein (LDL) levels. Individuals with cancers exhibiting loss of PTEN expression demonstrated lower mean blood LDL concentrations than individuals with colorectal cancers retaining PTEN expression (p = 0.05). Lower blood LDL levels may correlate with a generally more healthy diet and lifestyle, but the relevance of this observation is unclear and would benefit from further investigation.

*PIK3CA *mutations in exons 8, 9 and 20 were observed in 7.0% of cancers. This frequency is at the lower end of the range of previously reported frequencies of 10-20% [3-6] and may be due to the restricted region of analysis used (exons 8, 9 and 20). It has been reported that 70-80% of all *PIK3CA *mutations are located in the helical and kinase domains in these 3 exons [2,3,7] and as such, restricting analysis to these regions may have led to some mutations in other exons not being identified. In our previous analyses we also observed that 22% of the cancers in our study cohort possessed *K-RAS *mutations, including a previously unreported double mutation in codons 19 and 20 [20,26]. This frequency is lower than the 30-40% expected prevalence for mutations in this gene. Several observations suggest that these low mutation frequencies are accurate for this CRC cohort and not the result of technical shortcomings. First, our previous analysis of *BRAF *mutations in this same sample set demonstrated a higher than expected prevalence of this mutation (15.9%). Secondly, our analyses identified that 17% of the tumours in our dataset demonstrated an MSI phenotype, a level slightly above the expected ~15% prevalence of microsatellite instability in CRC [22]. As the same DNA samples were used across all analyses, these observations suggest that the observed low mutation frequencies in *PIK3CA *and *K-RAS *are a characteristic of this study cohort.

Fewer than 10% of samples demonstrated *PIK3CA *mutations, therefore analysis of dietary factors was not performed due to the calculated low power of the statistical testing. *PIK3CA *mutations were more frequent in CRC from females (p = 0.04). This has been demonstrated previously in another report analysing a Caucasian European population (Italian) [4], but not in a study of a Middle Eastern population [3]. These discrepancies in analyses of different population types may suggest a correlation between local environment, diet and *PIK3CA *mutation.

Analysis of PTEN expression negative tumours stratified by location demonstrated that proximally located cancers were more frequently of advanced Dukes' stage and distal colonic/rectal cancers were more frequently of less advanced Dukes' stage (p = 0.02). Additionally, proximal cancers lacking PTEN expression were associated with poor differentiation (p < 0.01). Stratification of PTEN negative cancers by stage and location also identified an increased mean non-starch polysaccharide consumption in those individuals with advanced stage cancers lacking PTEN expression (p = 0.01). This potentially interesting observation requires confirmation in future larger studies. Taken together, these data demonstrate the heterogeneity in the associations of cancers lacking PTEN expression: loss of PTEN expression in colorectal cancers of different locations have distinct clinicopathological features and potential dietary associations.

Analysis of co-occurrences of mutations in the PI3K and MAPK signalling pathways demonstrated that unlike *BRAF *and *K-RAS *mutations, acting as mutually exclusive mechanisms of activation of the same signalling pathway, oncogenic *PIK3CA *activation and PTEN loss are not equivalent. These two events share limited redundancy, indicating that both provide independent growth advantages for cancer cells. Mutations in *BRAF *and *PTEN *were associated: four adenocarcinomas had *PTEN *mutations, mostly in poly-A tracts, and all 4 CRC were MSI+ and proximally located, typical of defective DNA mismatch repair tumours.

A strength of the current study is the use of prospective data collected before disease onset, as well as employment of 7-day diaries for dietary assessment, a method shown to estimate diet more accurately than food frequency questionnaires [27]. This report greatly expands the limited current data describing alterations in PI3K/AKT pathway components in colorectal cancer and dietary intakes. Adjustment for confounding variables in the statistical testing was not performed. Such testing using logistic regression methods, performed on low sample sets has been described to lead to systematic bias (i.e. away from null), and overestimation of odds ratios [28]. In order to prevent overestimation of dietary risk factors, this testing was not performed on the relatively small sample sizes available.

This report is one of the first to analyse *PTEN *and *PIK3CA *mutation and PTEN expression in colorectal adenomas, albeit in a low number of cases. *PIK3CA *was mutated in a single adenoma (6.3%). Two adenomas (14.3%) demonstrated loss of PTEN expression. These data demonstrate that *PIK3CA *mutation and loss of PTEN expression can occur during adenoma formation. This confirms previous data describing *PIK3CA *mutations in adenomas [2,29]. This report is one of the first to analyse PTEN expression in colorectal adenomas. However, the small sample size indicates that the findings should be interpreted with caution.

## Conclusions

This study is one of the largest to date analysing PTEN expression in colorectal cancers, showing association of this with Dukes' stage and colorectal location, indicating a worse prognosis of PTEN expression negative cancers of the proximal colon that show poor differentiation and advanced Dukes' stage. Furthermore, mutations in the *PTEN *gene, although likely to be rare in unselected colorectal cancers, are associated with MSI+ and *BRAF *mutations, whereas loss of PTEN expression is not and may be due mostly to other mechanisms. These data describe the assessment of associations between dietary factors and loss of PTEN expression in colorectal cancers, suggesting that general loss of PTEN expression is independent of any specific dietary influences. Furthermore, increased signalling mediated by the PI3K/AKT pathway, achieved via mutations in *PIK3CA *or loss of PTEN expression are not mutually exclusive, suggesting that independent growth advantages are provided by these two cancer promoting changes in colorectal cancer.

## Competing interests

The authors declare that they have no competing interests.

## Authors' contributions

AN performed the sequencing analyses, statistical testing, composed the manuscript and assessed PTEN expression status. PNM contributed to manuscript design. LJG performed the MSI analyses. RYB obtained access to and distributed the human tissue samples. RNL and AM compiled and provided the dietary data. MJA contributed to manuscript preparation, supervision of the project, study design and assessed PTEN expression status. JCC, LK and LH contributed to the PTEN immunohistochemistry. All authors read and approved the manuscript.

## Pre-publication history

The pre-publication history for this paper can be accessed here:

http://www.biomedcentral.com/1471-2407/11/123/prepub

## References

[B1] Samuels Y Velculescu VE Oncogenic mutations of PIK3CA in human cancers Cell Cycle 2004 3 10 1221 1224 10.4161/cc.3.10.1164 15467468

[B2] Samuels Y Wang Z Bardelli A Silliman N Ptak J Szabo S Yan H Gazdar A Powell SM Riggins GJ High frequency of mutations of the PIK3CA gene in human cancers Science 2004 304 5670 554 10.1126/science.1096502 15016963

[B3] Abubaker J Bavi P Al-Harbi S Ibrahim M Siraj AK Al-Sanea N Abduljabbar A Ashari LH Alhomoud S Al-Dayel F Clinicopathological analysis of colorectal cancers with PIK3CA mutations in Middle Eastern population Oncogene 2008 27 25 3539 3545 10.1038/sj.onc.1211013 18193083

[B4] Benvenuti S Frattini M Arena S Zanon C Cappelletti V Coradini D Daidone MG Pilotti S Pierotti MA Bardelli A PIK3CA cancer mutations display gender and tissue specificity patterns Hum Mutat 2008 29 2 284 288 10.1002/humu.20648 18022911

[B5] Campbell IG Russell SE Choong DY Montgomery KG Ciavarella ML Hooi CS Cristiano BE Pearson RB Phillips WA Mutation of the PIK3CA gene in ovarian and breast cancer Cancer Res 2004 64 21 7678 7681 10.1158/0008-5472.CAN-04-2933 15520168

[B6] Velho S Oliveira C Ferreira A Ferreira AC Suriano G Schwartz S Jr Duval A Carneiro F Machado JC Hamelin R The prevalence of PIK3CA mutations in gastric and colon cancer Eur J Cancer 2005 41 11 1649 1654 10.1016/j.ejca.2005.04.022 15994075

[B7] Zhao L Vogt PK Class I PI3K in oncogenic cellular transformation Oncogene 2008 27 41 5486 5496 10.1038/onc.2008.244 18794883 PMC2757120

[B8] Chang JG Chen YJ Perng LI Wang NM Kao MC Yang TY Chang CP Tsai CH Mutation analysis of the PTEN/MMAC1 gene in cancers of the digestive tract Eur J Cancer 1999 35 4 647 651 10.1016/S0959-8049(98)00411-0 10492641

[B9] Danielsen SA Lind GE Bjornslett M Meling GI Rognum TO Heim S Lothe RA Novel mutations of the suppressor gene PTEN in colorectal carcinomas stratified by microsatellite instability- and TP53 mutation-status Hum Mutat 2008 18781614 10.1002/humu.20860

[B10] Dicuonzo G Angeletti S Garcia-Foncillas J Brugarolas A Okrouzhnov Y Santini D Tonini G Lorino G De Cesaris M Baldi A Colorectal carcinomas and PTEN/MMAC1 gene mutations Clin Cancer Res 2001 7 12 4049 4053 11751500

[B11] Goel A Arnold CN Niedzwiecki D Carethers JM Dowell JM Wasserman L Compton C Mayer RJ Bertagnolli MM Boland CR Frequent inactivation of PTEN by promoter hypermethylation in microsatellite instability-high sporadic colorectal cancers Cancer Res 2004 64 9 3014 3021 10.1158/0008-5472.CAN-2401-2 15126336

[B12] Nassif NT Lobo GP Wu X Henderson CJ Morrison CD Eng C Jalaludin B Segelov E PTEN mutations are common in sporadic microsatellite stable colorectal cancer Oncogene 2004 23 2 617 628 10.1038/sj.onc.1207059 14724591

[B13] Wang ZJ Taylor F Churchman M Norbury G Tomlinson I Genetic pathways of colorectal carcinogenesis rarely involve the PTEN and LKB1 genes outside the inherited hamartoma syndromes Am J Pathol 1998 153 2 363 366 10.1016/S0002-9440(10)65579-4 9708796 PMC1852980

[B14] Guanti G Resta N Simone C Cariola F Demma I Fiorente P Gentile M Involvement of PTEN mutations in the genetic pathways of colorectal cancerogenesis Hum Mol Genet 2000 9 2 283 287 10.1093/hmg/9.2.283 10607839

[B15] Zhou XP Loukola A Salovaara R Nystrom-Lahti M Peltomaki P de la Chapelle A Aaltonen LA Eng C PTEN mutational spectra, expression levels, and subcellular localization in microsatellite stable and unstable colorectal cancers Am J Pathol 2002 161 2 439 447 10.1016/S0002-9440(10)64200-9 12163369 PMC1850747

[B16] Frattini M Saletti P Romagnani E Martin V Molinari F Ghisletta M Camponovo A Etienne LL Cavalli F Mazzucchelli L PTEN loss of expression predicts cetuximab efficacy in metastatic colorectal cancer patients Br J Cancer 2007 97 8 1139 1145 10.1038/sj.bjc.6604009 17940504 PMC2360431

[B17] Molinari F Martin V Saletti P De Dosso S Spitale A Camponovo A Bordoni A Crippa S Mazzucchelli L Frattini M Differing deregulation of EGFR and downstream proteins in primary colorectal cancer and related metastatic sites may be clinically relevant Br J Cancer 2009 100 7 1087 1094 10.1038/sj.bjc.6604848 19293803 PMC2669991

[B18] Parkin DM Bray F Ferlay J Pisani P Global cancer statistics, 2002 CA Cancer J Clin 2005 55 2 74 108 10.3322/canjclin.55.2.74 15761078

[B19] Cummings JH Bingham SA Diet and the prevention of cancer BMJ 1998 317 7173 1636 1640 9848907 10.1136/bmj.317.7173.1636 PMC1114436

[B20] Naguib A Mitrou PN Gay LJ Cooke JC Luben RN Ball RY McTaggart A Arends MJ Rodwell SA Dietary, lifestyle and clinicopathological factors associated with BRAF and K-ras mutations arising in distinct subsets of colorectal cancers in the EPIC Norfolk study BMC Cancer 2010 10 99 10.1186/1471-2407-10-99 20233436 PMC2847960

[B21] Li VS Wong CW Chan TL Chan AS Zhao W Chu KM So S Chen X Yuen ST Leung SY Mutations of PIK3CA in gastric adenocarcinoma BMC Cancer 2005 5 29 10.1186/1471-2407-5-29 15784156 PMC1079799

[B22] Soreide K Janssen EA Soiland H Korner H Baak JP Microsatellite instability in colorectal cancer Br J Surg 2006 93 4 395 406 10.1002/bjs.5328 16555243

[B23] Dahia PL FitzGerald MG Zhang X Marsh DJ Zheng Z Pietsch T von Deimling A Haluska FG Haber DA Eng C A highly conserved processed PTEN pseudogene is located on chromosome band 9p21 Oncogene 1998 16 18 2403 2406 10.1038/sj.onc.1201762 9620558

[B24] Klose RJ Bird AP Genomic DNA methylation: the mark and its mediators Trends Biochem Sci 2006 31 2 89 97 10.1016/j.tibs.2005.12.008 16403636

[B25] Garcia JM Rodriguez R Silva J Munoz C Dominguez G Silva JM Carcereny E Provencio M Espana P Bonilla F Intratumoral heterogeneity in microsatellite alterations in BRCA1 and PTEN regions in sporadic colorectal cancer Ann Surg Oncol 2003 10 8 876 881 10.1245/ASO.2003.02.004 14527905

[B26] Naguib A Wilson CH Adams DJ Arends MJ Activation of K-RAS by co-mutation of codons 19 and 20 is transforming J Mol Signal 2011 6 2 10.1186/1750-2187-6-2 21371307 PMC3056876

[B27] Day N McKeown N Wong M Welch A Bingham S Epidemiological assessment of diet: a comparison of a 7-day diary with a food frequency questionnaire using urinary markers of nitrogen, potassium and sodium Int J Epidemiol 2001 30 2 309 317 10.1093/ije/30.2.309 11369735

[B28] Nemes S Jonasson JM Genell A Steineck G Bias in odds ratios by logistic regression modelling and sample size BMC Med Res Methodol 2009 9 56 10.1186/1471-2288-9-56 19635144 PMC2724427

[B29] Velho S Moutinho C Cirnes L Albuquerque C Hamelin R Schmitt F Carneiro F Oliveira C Seruca R BRAF, KRAS and PIK3CA mutations in colorectal serrated polyps and cancer: primary or secondary genetic events in colorectal carcinogenesis? BMC Cancer 2008 8 255 10.1186/1471-2407-8-255 18782444 PMC2553419

